# Miscellany

**Published:** 1890-12

**Authors:** 


### Miscellany.

THE Christmas edition of the *Cosmopolitan Magazine* is 100,000 copies. The order, as originally given to the printers, was for 85,000 copies, but while on the press it was thought advisable to increase the number to 100,000.

It contains a feature never before attempted by any magazine, consisting of 123 cartoons from the brush of Dan Beard, the now famous artist, who did such wonderful illustrations in Mark Twain's book, "The Yankee at the Court of King Arthur."

These cartoons are placed at the bottom of each page of the magazine, and take for their subject, "Christmas during the Eighteen Centuries of the Christian Era," with variations, showing the way in which we modern Christians carry out some of the chief texts of the Christian Gospel.

Above, and at each side of the page, is a quaint border, the whole effect being novel and extremely pleasing, and with the unusually varied table of contents, will make such a Christmas number as is worthy to go into more than 100,000 households.

The frontispieces of the *Cosmopolitan* have of late become noted for their beauty, some of them having as much as four printings. That for Christmas, while in but two printings, is not behind anything that has preceded it in artistic merit.

An excellently illustrated article is one on Teapots, by Eliza Ruhamah Scidmore. Literary Boston is treated with numerous portraits, and an article which comes with the ninetieth birthday of Von Moltke, sketches the life of the great Field-Marshal in an interesting way, and is by Gen. James Grant Wilson. Elizabeth Bisland has one of her charming articles.

*The Christmas issue contains 228 illustrations*, nearly double the number that have ever appeared in any illustrated magazine.

CONTENTS FOR DECEMBER.

Away on the Mountain, Wild and Bare (Frontispiece) ; The Passion Play at Oberammergau. Illustrated. Elizabeth Bisland ; The Race. (Poem.) George Edgar Montgomery ; The Cruise of the "Sonoma." Illustrated. T. H. Stevens ; Collections of Teapots. (Illustrated.) Eliza Ruhamah Scidmore ; The Army of

Japan. (Part II.) Illustrated. Arthur Sherburne Hardy; Hymn. (Poem.) John W. Weidemeyer; Field Marshal von Moltke. Illustrated. James Grant Wilson; Mrs. Pendleton's Four-In-Hand. Illustrated. Gertrude Franklin Atherton; Literary Boston. Illustrated. Lilian Whiting; Equanimity. (Poem.) William Wheeler; A Famous Fireplace. Illustrated. Herbert Pierson; The Birds of Nazareth. (Poem.) Illustrated. Elizabeth Akers; The Pursuit of the Martyns. (Part II.) Illustrated. Richard Malcolm Johnson; Hylas. (Poem.) Marion M. Miller; Review of Current Events, Murat Halstead; Social Problems, Edward Everett Hale.

---

The Alvarenga Prize.— The College of Physicians of Philadelphia announces that the next award of the Alvarenga Prize, being the income for one year of the bequest of the late Señor Alvarenga, and amounting to about $180, will be made on July 14, 1891. Essays intended for competition may be upon any subject in medicine, and must be received by the Secretary of the College on or before May 1, 1891.

---

King's Journal Directory for 1891, containing a complete list of Medical, Dental, Pharmaceutical, Chemical, Microscopical, Sanitary, Veterinary and Medico-Legal Journals, both Home and Foreign, will be ready for delivery on or before January 1st next. Orders should be sent promptly, as the book is sold by subscription only. Price, fifty cents, post-paid. Address Dr. F. King, Publisher, P. O. Box 587, New York. The *Directory* will be sent to libraries and managers of advertising departments, *free*.

---

The *Baltimore Medical and Surgical Record* is the name of a new monthly journal of medicine and surgery, that has presented itself for the favor of the profession. The first number was issued in October, 1890, and contains forty-two double column large octavo pages. It is edited and managed by T. H. Graham, 1602 West Lauvale street, Baltimore, Md., and appears to have come to remain.

